# The importance of medical treatment before surgery in pleomorphic carcinoma of the lung: A case series study

**DOI:** 10.1016/j.ijscr.2021.106275

**Published:** 2021-08-04

**Authors:** Francesco Tormen, Federico Banchelli, Valentina Masciale, Antonino Maiorana, Uliano Morandi, Beatrice Aramini

**Affiliations:** a Division of Thoracic Surgery, Department of Medical and Surgical Sciences, University of Modena and Reggio Emilia, Modena, Italy; b Center of Statistic, Department of Medical and Surgical Sciences, University of Modena and Reggio Emilia, Italy; c Institute of Pathology, Department of Medical and Surgical Sciences, University of Modena and Reggio Emilia, Italy

**Keywords:** Pleomorphic, Carcinoma, Lung cancer, Rare tumor, NSCLC

## Abstract

**Introduction and importance:**

Pleomorphic carcinoma of the lung is a rare malignant epithelial tumor. Due to its rarity, its clinicopathological characteristics are not clear, and there is no defined therapeutic path for this type of tumor.

**Case presentation:**

We retrospectively analyzed the medical and pathological reports of 8 patients who underwent surgical resection for pleomorphic carcinoma between 2007 and 2010.

**Clinical discussion:**

Eight patients were analyzed (7 males and 1 female, mean age 60). All patients underwent CT scans, and the average diameter of the nodules was 56 mm. Four patients were also investigated with FDG-PET with hypermetabolic activity in all four cases. In four patients, the carcinomatous component was adenocarcinoma (all with sarcomatoid component of spindle cell and giant cell carcinoma), although in two patients, it was squamous cell carcinoma (one with spindle cell and one with giant cell). In the two remaining patients, one showed a non-small cell carcinoma with giant cell carcinoma, and the other was a non-small cell carcinoma and squamous cell carcinoma with spindle and giant cell carcinoma. All cases were treated with surgical resection. Only two patients underwent neoadjuvant chemotherapy. At the time of data analysis, only one patient treated with neoadjuvant chemotherapy was alive.

**Conclusion:**

The prognosis for these patients with a diagnosis of pleomorphic carcinoma undergoing surgery is generally better than those not treated with surgical resection, however the survival remains poor. Although with low number of patients, our research would suggest to consider neoadjuvant chemotherapy an appropriate approach for improving the outcomes before surgery.

## Highlights

• Pleomorphic carcinoma of the lung
• Rare malignant epithelial tumor
• Clinicopathological characteristics unclear
• Not defined therapeutic path
• Neoadiuvant chemotherapy improves the prognosis.

## 1 Introduction

Pleomorphic carcinoma (PC) of the lung is a highly rare malignant tumor. According to the World Health Organization (WHO) classification of lung tumors, it is a poorly differentiated non-small cell carcinoma (NSCC) in which can be found a squamous cell carcinoma, adenocarcinoma, or undifferentiated NSCC that contains at least 10% spindle and/or giant cells, or a carcinoma consisting only of spindle and giant cells. Its incidence ranges from 0.1% to 0.4% of all lung cancers and generally occurs in males with smoking habits, with an average age at diagnosis of 60 years [1], [2], [3], [4].

The clinical course of PC is more aggressive than other non-small cell lung cancers (NSCLCs). This may be caused by the advanced stage of the disease's diagnosis or because of the generally poor response to systematic chemotherapy. Furthermore, the clinicopathological characteristics of PC are not well known because of its rarity and the low number of studies on it [2], [4], [5], [6], [7], [8].

The lack of uniform diagnostic criteria makes the diagnosis of PC problematic. There are no characteristic features in chest radiographs that can assist in the discrimination of PC lung carcinoma from other primary lung malignancies. The tumor appears to primarily affect the upper lobes and the lung periphery. However, some studies have shown that CT features of PC appear to be dictated by the epithelial component of the tumor. PC with an adenocarcinoma component tends to be located peripherally; PC with a squamous cell component has a strong predilection for the central location, and PC with a large cell carcinoma component tends to comprise bulky peripheral masses with multiple foci of necrosis. Nevertheless, all of these CT features are nonspecific, and they are not particularly different from those of ordinary NSCLC [1], [3], [9], [10].

Percutaneous needle biopsy generally does not add anything to the diagnosis because frequently it only shows a malignant epithelial component. Indeed, according to the WHO, the diagnosis of PC cannot be made on small biopsies or cytology. The use of antibodies against a large panel of cytokeratins helps to identify the epithelial component, which is almost always present. In the most recent WHO classification of lung carcinomas (2015), one new recommended aspect for diagnosis is molecular testing according to known genetic abnormalities associated with histologic components, such as EGFR mutation and ALK rearrangement in tumors with adenocarcinoma component [2], [9], [11].

It is reported in the literature that aggressive treatments, including preoperative chemoradiotherapy combined with surgical resection and post-operative adjuvant treatment, should be considered to prolong the life expectancy of patients with PC lung cancer. However, evidence of the benefit of chemotherapy for PC is also lacking, and it appears that palliative chemotherapy regimens commonly used for NSCLC are not effective at treating advanced pulmonary PC [3], [6], [12].

Surgery should be restricted to N0 patients, and patients with nodal disease should be considered for neoadjuvant therapy. Lymph node involvement seems to be, in fact, a significant negative prognostic factor in the survivability of these patients because survival curves showed that recurrences were more typical in patients with nodal disease involvement [1], [3], [13].

The aim of this article is to highlight a better outcome in terms of survival for the two patients who had undergone neoadjuvant chemotherapy and surgery, in contrast with patients who had undergone lung resection without neoadjuvant treatment. Although our pilot study was limited to a small number of cases, we believe that medical treatment before surgery improves the prognosis.

## 2 Methods

We retrospectively analyzed 8 cases of patients who had undergone surgery, from the beginning of 2007 until the end of 2010, at the Department of Thoracic Surgery of the University of Modena and Reggio Emilia, with subsequential anatomo-pathological diagnosis of PC carcinoma of the lung. Patients were analyzed for age at diagnosis, sex, smoking habit, and number of cigarettes per day (measured in pack/years), comorbidities, and symptoms. No drug history, no family history including any relevant genetic information, and no psychosocial history have been declared by each patients enrolled.

Chest CT scan was performed in all the patients, identifying the location and dimension of the lung lesion. Some of them also underwent FDG-PET scan or invasive diagnosis investigations such as transthoracic lung biopsy (TTLB) or fine-needle aspiration biopsy (FNAB) during a bronchoscopy exam, which provided the opportunity for a pre-surgery diagnosis.

We assessed whether patients were treated with neoadjuvant or adjuvant chemo–radio therapy. All patients underwent major lung resection (lobectomy with lymph nodes resection) by lateral thoracotomy at our Department of Thoracic Surgery. No morbidity or mortality have been occurred postoperatively. All surgical resection specimens were then analyzed by expert anatomopathologists. The tumors were classified according to the 2001 WHO classification: PC is defined as a poorly differentiated non-small cell carcinoma (NSCC) in which a squamous cell carcinoma, adenocarcinoma, or undifferentiated NSCC that contains at least 10% spindle and/or giant cells, or a carcinoma consisting only of spindle and giant cells, can be found. We recorded the findings of the two histological components, the presence of necrosis, lymphonodal invasion, and the infiltration of other structures such as pleura or vessels.

We also obtained information on patients' outcomes, recurrences of the lesion, the time interval from surgery to recurrence, death, and time from surgery to death. All clinical data were collected from medical records, pathologic reports, and internal hospital databases.

Data were described using the mean and range of continuous variables and by using absolute or percentage frequencies for categorical variables. The median survival time was estimated using a Kaplan-Meier curve. Analyses were carried out with the use of R 3.6.3 statistical software (The R Foundation for Statistical Computing, Wien).

This work has been reported in line with the SCARE criteria [14] and it is compliant with the PROCESS Guidelines [15].

## 3 Results

The patients' characteristics are listed in Table 1. Of the 8 patients, only one was a woman (12.5% of total cases); the patients were aged between 76 and 42 years, and the average age at diagnosis was 60 years. All except one were smokers, with an average consumption of 38 pack/years (ranging from 15 to 60 pack/years; considering 20 cigarettes per pack). Five patients presented with symptoms before receiving any treatment: one with cough, one with cough and fever, and three with hemoptysis. Only one of the patients presented with other carcinomatous diseases in their clinical history.

**Table 1:** Clinicopathological characteristics of 8 patients with pleomorphic carcinoma of the lung.

No of patient	Age	Sex	Symptoms	TNM	Stage	Smoking history	Location	Initial treatment	Relapse	Outcome	Survival from surgery (months)
1	75	M	Cough	T3N0M0	IIB	Yes	RLL	Surgical resection	Yes	Died	25
2	76	M	None	T2N1M0	IIB	No	RLL	Surgical resection	Yes	Died	3.7
3	64	M	Hemoptysis	T2N1M0	IIB	Yes	RUL	Surgical resection	Yes	Died	2.7
4	62	F	Cough, Fever	T2N0M0	IIA	Yes	RUL	Chemotherapy	No	Alive	130.0
5	64	M	Hemoptysis	T3N0M0	IIB	Yes	LUL	Surgical resection	Yes	Died	17.9
6	42	M	Hemoptysis	T1N1M0	IIB	Yes	RUL	Chemotherapy	Yes	Died	30
7	51	M	None	T2N0M0	IB	Yes	LLL	Surgical resection	Yes	Died	10.2
8	52	M	None	T4N2M0	IIIB	Yes	RUL	Surgical resection	Yes	Died	1.2

All 8 cases were tested with CT chest scan; the dimension of the lesions ranged between 20 and 96 mm with an average nodule diameter of 56 mm. The locations of the lesions were as follows: 5 were in the upper lobe, with 4 on the right side and 1 on the left side; 2 were in the right lower lobe, and 1 in the left lower lobe. Four patients completed the imaging diagnosis with FDG-PET, and all of them had documented hypermethabolic capitation of the known lesion of the lung. Five of them were also tested with other invasive diagnosis methodologies: three nodules were investigated with FNBA and two with TTLB. Only three of them received a pre-surgical diagnosis. Nodules were diagnosed as adenocarcinoma (2 out of 4), squamous cell carcinoma, and bronchogenic carcinoma.

Patients' perspectives have been thought to be good after surgical treatment, for the asportation of the malignant nodule. Infact, of the 8 patients, only two patients received neoadjuvant chemotherapy: one patient (patient n°4) was treated with 6 cycles of chemotherapy with cisplatin–gemcitabine (CDDP-GEM) and one (patient n°6) with 3 cycles of CDDP-GEM. After surgical resection, only two patients received adjuvant chemotherapy and four received adjuvant radiotherapy (patient n° 4 was not one of them; instead, patient n°6 received adjuvant radiotherapy).

The pathological features of the surgical samples are listed in Table 2. For four samples, the carcinomatous component was adenocarcinoma, and all of them had a sarcomatoid component of spindle cell and giant cell carcinoma; two were squamous cell carcinoma, one with spindle cell, and one with giant cell. The two remaining samples were one being a non-small cell carcinoma with giant cell carcinoma and the other was a non-small cell carcinoma and squamous cell carcinoma with spindle and giant cell carcinoma. All of the samples appeared to have infiltrations to other tissues: 4 nodules infiltrated visceral pleura and vessels, 1 nodule infiltrated visceral and parietal pleura, 1 infiltrated only visceral pleura, 1 only vessels, and the last 1 infiltrated both the visceral and parietal pleura and vessels. Four of 8 contained necrotic areas. In 4 cases, lymphnodal involvement was observed. Regarding the pathological stage of the tumor, one case was stage IB, one was IIA, and the remaining sex were stage IIB. All but one patient (patient n° 4) had recurrence (87.5%), which occurred from 1.1 to 5.4 months after surgery (average 3.1 months). Seven patients died at the time of our analysis. Patients died after an average of 5.1 months from relapsing (range from 0.1 to 21 months), and the median survival time after surgery was 19.3 months (range 1.2–130.0 months).

**Table 2:** Pathological findings.

No of patients	Carcinomatous component	Sarcomatoid component	Infiltration	Necrosis	Lymphonodal involvement
1	Adenocarcinoma	Spindle cell and Giant cell	Visceral and parietal pleura	Yes	No
2	Squamous cell carcinoma	Spindle cell	Visceral pleura and vessel	No	Yes
3	Adenocarcinoma	Spindle cell and Giant cell	Visceral pleura and vessels	No	Yes
4	Adenocarcinoma	Spindle cell and Giant cell	Visceral pleura and vessels	No	No
5	Squamous cell carcinoma	Giant cell	Visceral pleura	Yes	No
6	Non-small cell carcinoma and squamous cell carcinoma	Spindle cell and Giant cell	Vessels	No	Yes
7	Non-small cell carcinoma	Giant cell	Visceral pleura and vessels	Yes	No
8	Adenocarcinoma	Spindle cell and Giant cell	Visceral and parietal pleura and vessels	Yes	Yes

## 4 Discussion

PC of the lung is a poorly differentiated non-small cell carcinoma (NSCC) in which can be found a squamous cell carcinoma, adenocarcinoma, or undifferentiated NSCC that contains at least 10% spindle and/or giant cells, or a carcinoma consisting only of spindle and giant cells (Table 3). PC is rare, accounting for 0.1–0.4% of all lung neoplasms and it is, therefore, rather difficult to diagnose. Further, prognostic factors and therapeutic strategies have yet to be found; hence, the prognosis for patients with PC is poor (Table 3). In our retrospective study, we analyzed 8 patients with post-surgical diagnosis of PC in relation to their neoadjuvant treatment in an attempt to highlight the outcome of two of them who had undergone pre-surgical chemotherapy.

**Table 3:** Review of the literature.

Autor/year	Journal	Number of patients enrolled	Sex (M/F)	Treatment (n of patients)	Alive/death	Average survival (months)
N. F. Fishback et al. (1994)	Cancer	78	57/21	Surgery (57)	9/69	23
Y-L Chang et al. (2001)	Lung Cancer	16	13/3	Surgery (7)/ CT (9)	0/16	5 (surgery); 2,7 (chemotherapy)
*G. Rossi* et al. (2003)	The American Journal of Surgical Pathology	75	68/7	Surgery	26/49	19
F. Raveglia et al. (2004)	The Society of Thoracic Surgeons	20	14/6	Surgery	4/16	13,8
T. H. Kim et al. (2004)	Radiology	10	7/3	Surgery (all) / Adjuvant CT (3) / Adjuvant RT (1)	4/6	3
T. S. Kim et al. (2004)	American Journal of Radiology	30	27/3	Surgery		
H. M. Bae et al. (2007)	Lung Cancer	13	9/4	Neoadjuvant CT (2), Surgery (8), Adjuvant CT (3), CT (5)	4/13	5
T. Yuki et al. (2007)	The Journal of Thoracic and Cardiovascular Surgery	45	41/4	Surgery		2,6
S. Yamamoto et al. (2007)	European Journal of Cardio-Thoracic Surgery	21	18/3	Surgery (all) / Adjuvant CT (9) / Adjuvant RT (4)	18/3	7,5
K Ito et al. (2009)	Lung Cancer	22	19/3	Surgery (15) / Adjuvant CT (7) / Adjuvant RT (2) / CT (3) / RT (4)	9/13	
T. Mochizuki (2008)	The American Journal of Surgical Pathology	70	57/13	Surgery (all)	29/41	11
K. Kaira et al. (2010)	Journal of Thoracic Oncology	17	13/4	Surgery (9) / CT (6) / RT (3) / None (1)	8/9	5,5
K. Okuda (2017)	Journal of Thoracic Disease	24	17/7	Neoadjuvant CT (15) / Surgery (all) / Adjuvant CT (5)	12/10	

CT, Chemotherapy; RT, Radiotherapy.

Unless our studied population comprised a low number of patients, it was composed of predominantly male subjects (87,5%), with a mean age at diagnosis of 60 years and smoking habits, in accordance with the literature [1], [4], [7].

Diagnosis of PC was not possible before surgery for any of the patients who were examined. As other studies have suggested, lesions are generally found in the upper lobes (62.5% of cases). Kim et al. [10] examined CT scans of 30 patients with PC and found that 23 out of 30 cases had a localization in the upper lobes. In addition, Kim et al. [9] showed that PC tends to prefer peripherical localization.

It is also indicated in the literature that FDG-PET can assist in the diagnosis of PC because the maximum standardized uptake value (SUVmax) tends to be clearly higher than other histological types of NSCLCs [8], [16]. Ito et al. [17] analyzed 22 cases of PC, with 12 of them demonstrating a higher level of the SUV of the primary lesion; their SUV values were increased compared to other NSCLC lesions, suggesting that FDG-PET is a valid investigation method to differentiate PC from other common NSCLCs. Four of the eight cases studied by us were subjected to an FDG-PET investigation, and all of them showed a hypermetabolic uptake, unless we were unable to find SUVmax values. Of the cases that underwent invasive diagnostic investigation, none had a pre-surgical diagnosis of PC.

PC has uncleared clinical characteristics due to the difficulty in obtaining a sufficiently large number of cases for analysis. Some of the consequences are the unclear preoperative strategy for PC and the lack of therapeutic strategies [1], [5], [6].

However, it is reported in the literature that patients not treated with surgery have a poor prognosis and that a complete resection with an appropriate follow-up therapy is an important factor for improving the outcome of patients with PC [3], [16], [17].

Our work aimed to analyze a group of patients who had undergone surgery. However, due to the rarity of PC, the number of patients was small. This represents the major limitation of our study. Although all patients were treated with major surgical lung resection (lobectomy), we determined that two patients had received neoadjuvant chemotherapy before surgery: patient n°4 was treated with 6 cycles of CDDP-GEM, and patient n°6 with 3 cycles of the same chemotherapy. Patient n°4 was still alive at the time of data collection, and although patient n°6 had died by that stage, the dimension of its lesion at the time of histological diagnosis was the smallest of all cases (20 mm). Patient n°6's lesion was the only lesion categorized as a T1 lesion in the histopathological analysis; otherwise, all the others were T2 or superior. Further, in the case of n°6, the lesion did not invade any structure except for the vessels and did not present any necrosis site. Mochizuki et al. [7] observed tumor necrosis in 89% of the studied cases and suggested that necrosis is a histologic prognostic factor for a poorer outcome of PC. Necrosis might be a pathological manifestation of rapidly proliferating tumor cells that have overgrown their blood supplies, leading to cell death. Both n°4 and n°6 did not present necrosis sites, although case n°4 presented lymphonodal involvement. Raveglia et al. [1] examined 20 patients who had been treated surgically and with a PC diagnosis: no patients with lymphonodal invasion survived. Rossi et al. [4] and Fishback et al. [5] also suggested that nodal invasion tends to shorten the overall survival of patients, potentially explaining the poor outcome of patient n°6 in contrast with patient n°4, who did not present with nodal involvement.

There are only relatively few studies in the literature examining the outcome of patients with PC who had been treated with neoadjuvant chemotherapy. Bae et al. [12] showed that the regimens commonly used for NSCLC are not effective for PC; they used different combinations of chemotherapeutic drugs, such as gemcitabine and cisplatin; however, despite these being viable treatments for NSCLC, they lead to only a relatively poor response in advanced PC. Other researchers have obtained different results [8], [16]; however, Okuda et al. [16] examined 5 patients who had undergone neoadjuvant chemotherapy, with the combination of cisplatin and vinorelbine being found to be effective against PC (2 courses before surgery). Kaira et al. [8], instead, determined that the combination of CDDP and GEM was effective in stage III disease, suggesting that chemotherapy commonly used for NSCLC may be particularly beneficial for advanced PC.

In conclusion, this study highlights the outcomes of two patients who had undergone neoadjuvant chemotherapy. The outcome of one patient (case n°4), who survived more than 10 years after surgical resection of his lung lesion, and the pathological findings of case n° 6, whose lesion presented on histological examination with a small dimension and an absence of intra-tumoral necrosis, might suggest the beneficial role of pre-surgical chemotherapy. Although surgical resection of the lesion appears to be essential for improving the outcome of patients with PC of the lung, neoadjuvant chemotherapy may be even more useful for enhancing these patients' prognoses. However, it is not yet clear in the literature which type of chemotherapy or combination of chemotherapeutic drugs should be used before and after surgery; however, we would like to focus on the possibility of treatment combinations, firstly set on a neoadiuvant chemotherapy approach, which seems to improve the outcomes after surgery. Further studies in a larger population need to be conducted to confirm our observations.

## Abbreviations

CDDP-GEM: Cisplatin-Gemcitabine
LUL: left upper lobectomy
LLL: left lower lobectomy
NSCLC: non-small cell lung cancer
PC: pleomorphic carcinoma
RUL: right upper lobectomy
RLL: right lower lobectomy
SUVmax: maximum standardized uptake value

## Sources of funding

NA

## Ethical approval

This study, involving human subjects and human data, has been performed in accordance with the Declaration of Helsinki and has been approved by the Ethics Committee at the University Hospital of Modena, MODENA, Italy, on 14 July 2020, Study n. 621/2020/OSS*/AOUMO SIRER ID 326, Prot. N. AOU 0019715/20. Further information and documentation to support this is available to the Editor on request.

## Consent

Written informed consent was obtained from the patients alive at the moment of the study for publication of this case report and accompanying images. A copy of the written consent is available for review by the Editor-in-Chief of this journal on request.

## Author contribution

The idea for the manuscript was conceived in June 2020 by BA and was further developed by FT, and FB; AM was involved in histopathological diagnosis. BA, FT, and FB wrote the first draft of the manuscript. BA and UM have been involved in surgery and tissue collection, whereas FB and RD performed the statistical analysis. BA, FT, FB, VM, FB, AM, and UM reviewed the manuscript and were involved in its critical revision before submission. All authors have read and approved the final manuscript.

## Research registration

NA.

## Guarantor

Beatrice Aramini MD PhD.

## Provenance and peer review

Not commissioned, externally peer-reviewed.

## CRediT authorship contribution statement

Francesco Tormen: Writing- Original draft preparation; Federico Banchelli: Data curation and Statistical analysis; Valentina Masciale: Sources curation and Visualization; Antonino Maiorana: Supervisor, Investigation; Uliano Morandi: Supervisor; Beatrice Aramini: Revision draft preparation, Conceptualization, Methodology, Investigation. All authors have read and approved the final manuscript.

## Declaration of competing interest

None of the Authors has any competing interests.
